# Isolated Malleus Fracture from Sneezing: A Case Report

**DOI:** 10.7759/cureus.5037

**Published:** 2019-06-29

**Authors:** Nina W Zhao, Philip Perez, Jeffrey D Sharon

**Affiliations:** 1 Otolaryngology - Head and Neck Surgery, University of California - San Francisco, San Francisco, USA

**Keywords:** malleus fracture, conductive hearing loss, sneezing, ossiculoplasty

## Abstract

Isolated malleus fractures are an infrequent cause of hearing loss. Even more unusual is a fracture secondary to a sneeze. Here, we review the case of a 32-year-old man with the first surgically confirmed malleus fracture due to a suppressed sneeze, which was then successfully repaired with hydroxyapatite bone cement. We discuss the presentation, diagnosis, and management of this patient and review the literature on isolated malleus injuries.

## Introduction

Hearing loss from an isolated malleus fracture is an uncommon clinical entity. While malleus fractures can result from direct trauma, they are most often reported as a result of implosive forces from digital manipulation of the ear canal, penetrating trauma, or external blunt trauma [1]. Here, we present an unusual case of a malleus fracture due to a suppressed sneeze that presumably created a sudden explosive force on the tympanic membrane (TM). The injury was then successfully repaired with hydroxyapatite bone cement via a transcanal, endoscopic-assisted approach. This is a unique report of successful repair of a malleus fracture due to this very uncommon pathophysiologic mechanism.

## Case presentation

A 32-year-old man presented to our clinic with several months of left otalgia, aural fullness, tinnitus, and decreased hearing that briefly improved with auto-insufflation. He reported a longstanding habit of sneezing with his mouth closed while pinching his nose and had done this several months prior to presentation followed by sudden onset of his symptoms. At that time, he went to a local emergency department where he was told he may have a small TM perforation.

**Figure 1:**
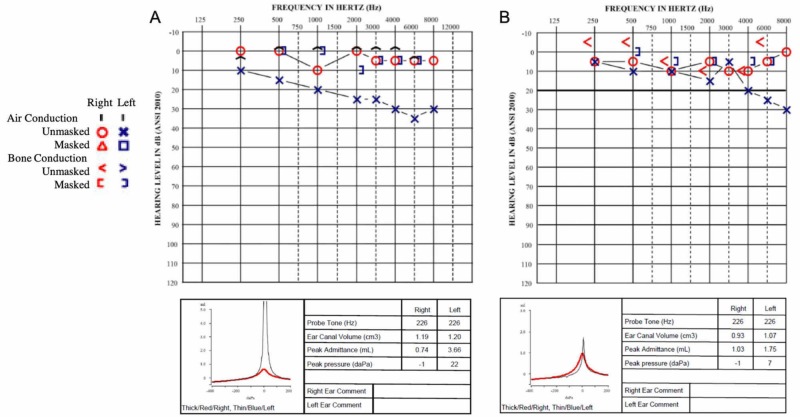
Preoperative and Postoperative Audiograms Preoperative (A) and two-month postoperative (B) audiograms and tympanometry, showing closure of air-bone gap and normalization of tympanometry.

On our initial exam, he was found to have normal TMs bilaterally with well-aerated middle ears. The Weber test with a 512 Hz tuning fork lateralized to the right ear, and the Rinne test was positive bilaterally. Audiometry was notable for normal right-sided hearing and mild conductive hearing loss on the left. The pure tone average (PTA) at 0.5, 1, 2, and 4 kHz was 22.5 dB (Figure 1A). Mean air-bone gap (ABG) at the same frequencies was 18.8 dB. The ABG ranged from 5 to 30 dB across all tested frequencies and was greatest at 6 kHz. Word recognition was excellent bilaterally. Tympanometry with a 226 Hz probe tone was normal on the right and hypermobile on the left (Figure 1A). Acoustic reflexes were absent in the left ipsilateral and right contralateral conditions. They were present in the right ipsilateral condition. In the left contralateral condition, the reflexes were elevated at 500 and 1000 Hz but absent at 2000 Hz.

**Figure 2:**
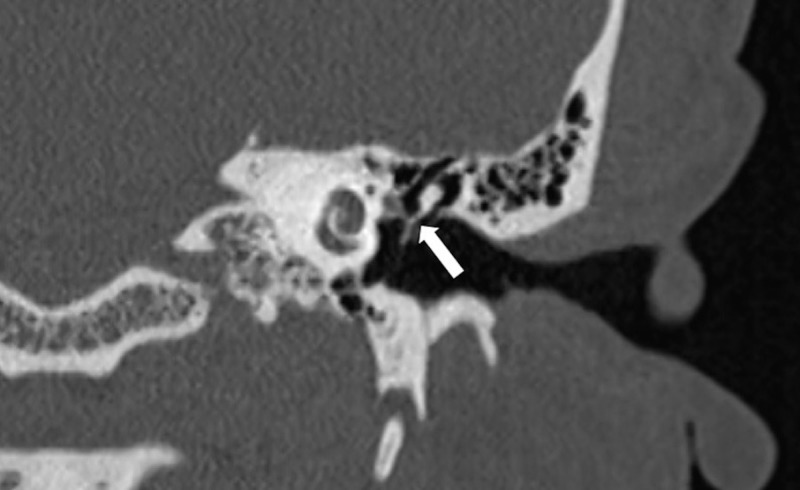
Preoperative Computed Tomography of the Left Temporal Bone Preoperative high-resolution computed tomography scan of the left temporal bone in the coronal plane demonstrating a nondisplaced transverse lucency of the malleus (white arrow).

**Figure 3:**
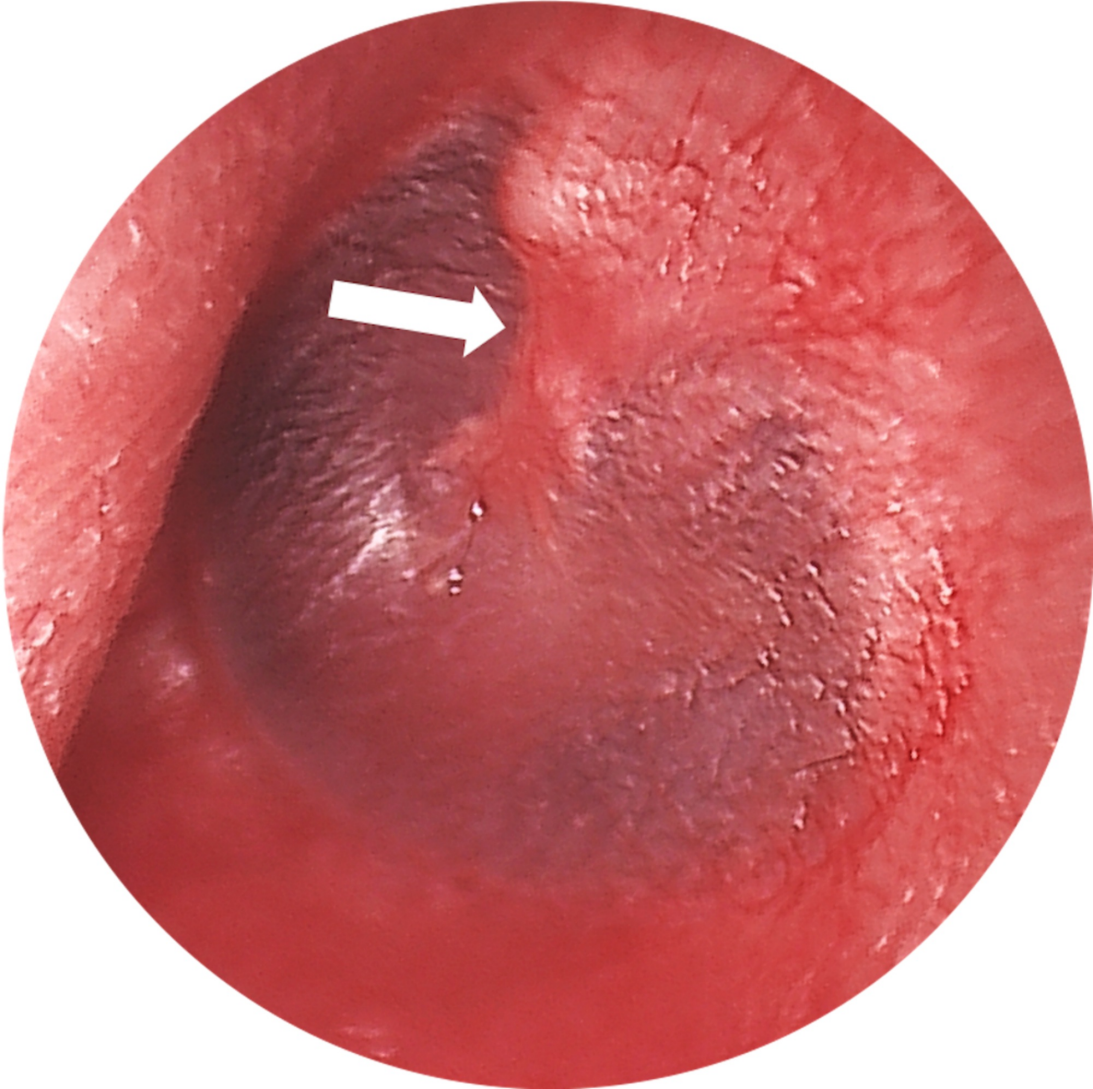
Preoperative Left Tympanic Membrane Preoperative endoscopic view of the left tympanic membrane demonstrating a subtle contour deformity of malleus (white arrow).

High-resolution computed tomography (CT) scan was initially read as normal. However, on further review, a non-displaced transverse lucency of the malleus handle was seen, consistent with a fracture (Figure 2). On repeat otoscopy, there appeared to be a small subtle contour deformity of the malleus (Figure 3).

**Figure 4:**
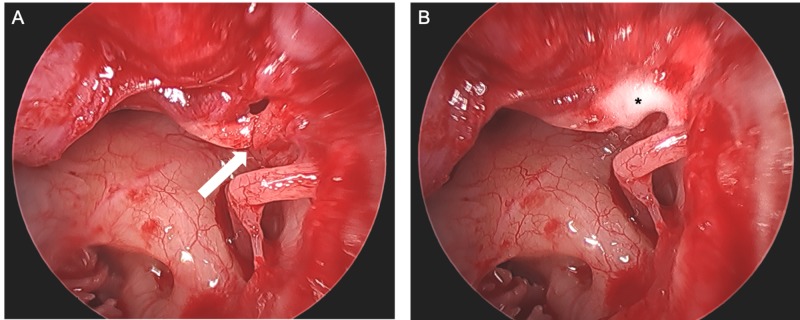
Intraoperative Left Malleus Fracture Intraoperative endoscopic view of the left middle ear demonstrating a fracture at the neck of the malleus (white arrow) before (A) and after (B) repair with hydroxyapatite (asterisk).

Following a discussion, the patient elected for exploratory tympanotomy. He was taken to operating room, and a left tympanoplasty was performed under general anesthesia with the assistance of an endoscope. After the tympanomeatal flap was raised, a fracture was discovered just below the neck of the malleus (Figure 4A). The remainder of the ossicles had a normal appearance and were mobile with an intact round window reflex. Mucosa and scar were cleared from around the fracture to expose 1-2 mm of dry bone superiorly and inferiorly. The fracture was reduced, and hydroxyapatite cement (OtoMimix; Gyrus ACMI, Inc., Southborough, MA, USA) was applied between the two mallear ends as well as along the denuded bone and allowed to set (Figure 4B). The strength of the repair was then tested with a microinstrument and the malleus appeared to move as a single unit. The reconstruction was stabilized with absorbable gelatin sponges (Gelfoam, Pfizer Inc., New York, NY, USA). The tympanomeatal flap was replaced and additional Gelfoam and antibiotic ointment was placed into the external auditory canal.

At two months post-surgery, the patient reported subjective improvement in hearing back to pre-injury levels. His Weber returned to midline, and his Rinne remained positive bilaterally. His repeat audiogram showed a PTA of 13.8 dB and ABG 10 dB on the left with intact acoustic reflexes bilaterally (Figure 1B). His TM compliances also normalized on tympanometry. He reported that he no longer closed his nose with sneezing.

## Discussion

The most common reported cause of malleus fractures is digital manipulation of the external auditory canal [1]. Other etiologies include direct instrumentation, head trauma, and, very rarely, barotrauma [2]. In our review of the literature, we found only one prior case in which an isolated malleus fracture was felt to be caused by a suppressed sneeze [3]. This patient had a left-sided hearing loss for 15 years. He did not have a history of significant noise trauma, head trauma, or infections, but did endorse a habit of pinching his nose while sneezing. On exam, he was found to have a posterior angulation of his distal malleus handle with an intact TM. No imaging was obtained, and the patient was referred for hearing aids. As a result, the diagnosis of a malleus fracture was never confirmed with surgery. Furthermore, symptom onset in that case was unclear. Conversely, in our patient, the symptoms occurred immediately after the insult, and the fracture was verified intraoperatively.

Ossicular injury after sneezing is rare. Most reported cases of hearing loss after sneezing are related to inner ear conditions, such as labyrinthine trauma and perilymphatic fistula [4, 5]. However, other isolated cases of middle ear injury have been reported. In 1975, Azem and Caldarelli discovered a stapes footplate fracture in a patient who developed sudden conductive hearing loss after sneezing. They performed a stapedectomy, and the patient’s hearing returned to normal six months postoperatively [6]. Whitehead reported three additional stapes footplate fractures in the setting of sudden sensorineural hearing loss, two of which were related to sneezing [7].

The malleus is thought to be an uncommon site of ossicular injury due to its relatively broad support within the middle ear compared to the other ossicles [8]. The malleus is supported by the TM and has strong ligamentous attachments through the anterior malleal ligament, the anterior, superior, posterior, and lateral suspensory ligaments, and the tensor tympani tendon. These ligaments are thought to be essential to diminishing pressure-induced displacements of the malleus as the tensor tympani itself does not have enough force to stabilize the TM-malleus complex against a hyperinflation of the middle ear [8]. We hypothesize that our patient’s malleus or malleolar ligaments may have been weakened over the years due to repeated inflation of the middle ear during suppressed sneezing.

Based on the recent literature, patients with isolated malleus fractures typically report sudden hearing loss, aural fullness, and tinnitus, with or without a clicking [9]. Vertigo is less common. Audiometric findings typically include a mild conductive hearing loss in the mid to high frequencies and a hypercompliant TM [1]. Similar to our case, ABG in patients with malleus fracture is usually larger in the higher frequencies compared with lower ones, which differs from other middle ear lesions such as ossicular fixation or otitis [10]. However, these fractures can be easily overlooked due to the relatively normal appearance of the TM. Therefore, thorough microscopic examination by an experienced clinician is necessary. CT may or may not reveal a fracture, especially if the fracture is nondisplaced and the image is not optimally formatted in the axis of the malleus. Still, most authors recommend the use of CT to aid in diagnosis and assess for other pathologies [11].

There are a variety of management options available for patients with an isolated malleus fracture. Some authors recommend a period of watchful waiting prior to surgical repair with surgery for patients with a persistent ABG or symptoms [10]. However, others agree that the condition is unlikely to heal spontaneously; therefore, surgery is the best option if the patient desires improvement [1, 11]. Techniques for surgical repair have included partial ossicular protheses, incus transposition grafts, malleus-to-incus osseous interposition grafts, and removal of the fractured segment [1, 10-12]. We were able to use bone cement with good short-term results though long-term follow-up is needed to assess for durability of the repair. Several other groups have used bone cement with varying degrees of success [13]. There are currently too few reports and no controlled studies to determine the superiority of any one of the techniques. At this time, choice of surgical repair is at the discretion of the surgeon in conjunction with patient preferences.

## Conclusions

We present the first surgically confirmed report of a malleus fracture due to a suppressed sneeze that was successfully repaired with hydroxyapatite bone cement. Malleus fracture after sneezing is rare but presents similarly to other causes, including sudden hearing loss and aural fullness. An elevated index of suspicion for these lesions is needed due to the subtle findings on physical exam and CT imaging. However, a conductive hearing loss with a hypercompliant TM can help in the diagnosis. Surgical repair is indicated in patients with a significant ABG or bothersome symptoms, and hydroxyapatite bone cement is a viable option.
